# Reliability of a Test for Assessment of Isometric Trunk Muscle Strength in Elderly Women

**DOI:** 10.1155/2019/9061839

**Published:** 2019-07-01

**Authors:** Marceli M. A. Mesquita, Marta S. Santos, Alan B. S. Vasconcelos, Clodoaldo A. de Sá, Luana C. D. Pereira, Ínea B. M. da Silva-Santos, Walderi M. da Silva Junior, Dihogo G. de Matos, Alan dos S. Fontes, Paulo M. P. Oliveira, Felipe J. Aidar, Josimari M. DeSantana, Iohanna G. S. Fernandes, Marzo E. Da Silva-Grigoletto

**Affiliations:** ^1^Department of Physical Education, Federal University of Sergipe, São Cristóvão, Brazil; ^2^Department of Physiology, Federal University of Sergipe, São Cristóvão, Brazil; ^3^Postgraduate Program in Health Sciences, Community University of the Region of Chapecó, Chapecó, Brazil; ^4^Department of Physiotherapy, Federal University of Sergipe, São Cristóvão, Brazil; ^5^Department of Sports Science, Exercise and Health of the Trás-os-Montes e Alto Douro University, Vila Real, Portugal; ^6^Department of Physiotherapy, Federal University of Sergipe, Lagarto, Brazil; ^7^Scientific Sport Association, Cordoba, Spain

## Abstract

**Objective:**

The aim of this study was to analyze the reproducibility of a protocol using the maximal isometric strength test of the trunk in elderly women aged above 60 years, without low back pain.

**Methods:**

Twenty-one physically inactive elderly women, who had not engaged in any activity or exercise program in the past three months, participated in the cross-sectional study that consisted of two days of evaluations for the maximal isometric strength of the extensor and flexor muscles of the trunk, with a 48 h interval between the sessions. A platform with fixed seating was used, which allowed the fixation of the hip and lower limbs, with a load cell connected to a linear encoder. To verify the reliability of the test, the interclass correlation coefficient, variation coefficient, minimum detectable difference (MDD), standard error of measurement, and Bland–Altman graphs were calculated.

**Results:**

No statistical difference was observed between the first and second evaluation, which indicates that there was no learning effect. Interclass correlation coefficient values were classified as very high and high for extensor (0.98) and flexor (0.86) muscles, respectively, besides low variation (9% for both muscle groups) and acceptable values for minimum detectable difference (extensors = 51.1 N, flexors = 48.9 N). In addition, the Bland–Altman analysis revealed low bias and values within the limits of agreement.

**Conclusion:**

It is concluded that the test of maximum isometric strength of the trunk in healthy and trained elderly people presents high reliability. These values proved to be reliable if performed in at least two evaluation sessions, which confirms the hypothesis of the authors by the consistency of the measurement test.

## 1. Introduction

Decreased muscle strength due to age is a determinant factor for the physical function in the elderly, which can lead to reduced functionality and performance and disability during daily life activities [1]. Previous studies investigating the relationship between the muscle strength and functional capacity in the elderly have focused on the peripheral musculature. However, more recent research has focused on changes related to trunk muscles, mainly due to their important role in performing activities of daily living (ADLs) and in terms of better functional performance [2, 3]; also the production of strength by the dorsal musculature constitutes an important parameter to evaluate the state of health of an individual [4].

Loss of functional capacity is associated with multiple factors; however, sarcopenia, characterized as the loss of muscle mass with consequent general functional decline leading to weakness, is the main contributor to this decline [5, 6]. As a consequence of sarcopenia, there is a reduction of maximal isometric strength, muscular power, and rate of strength development, that is, a reduced functional capacity during daily activities, such as walking, climbing stairs, squatting, or carrying something [7].

Strength assessment can also provide essential information on how the reduction of strength is related to the functional limitations of daily activities. However, the evaluation by isokinetic devices, despite being considered a gold standard for the measurement of muscle strength, is often not feasible due to the high cost of equipment and operational complexity [3, 8]. Therefore, the development of reliable and low-cost tests and protocols for evaluating the muscular strength of the trunk can facilitate the evaluation and monitoring of the force, especially in the elderly population. Thus, isometric dynamometry can be considered an accepted alternative tool to evaluate the maximum strength of the upper and lower limbs [9], as well as the trunk muscles [10]. Studies that investigated isometric trunk strength, assessed by dynamometry in the elderly, showed high to very high reliability for these measurements in test-retest studies [1, 10].

It should be noted, however, that relevant clinical aspects such as strength, balance, and force output are necessary to interpret the reliability of the isometric trunk test with the elderly population [11]. The inclusion of these measures allows for greater reliability of the method; therefore, the isometric dynamometry allows the measurement of muscle strength, whose decline as a result of aging generates incapacities to perform daily activities [12], besides being a safe tool for the evaluation of this population. This assessment instrument was used in a previous study [8] in order to assess the strength of the trunk in athletes and young people. However, no studies have been undertaken that used this instrument in the elderly.

Thus, the present study aimed to verify the reliability of an evaluation protocol using the maximal isometric strength test of the trunk in women older than 60 years [8]. Our hypothesis is that this protocol will demonstrate good reliability for the extensor and flexor muscles of the trunk, with a coefficient of variation (CV) within 10% of the mean.

## 2. Methods

### 2.1. Participants

The sample size was calculated using the G ∗ Power 3.1.9.2™ program and considering *α* = 0.05, *β* = 0.20, ratio of power correlation to null hypothesis of 0.35, and ratio of power correlation to alternative hypothesis of 0.80 [13]. At least 20 participants were needed for the study. Considering a potential loss of 20%, 25 older adults were recruited. Four of them missed the second testing day due to personal problems. Therefore, 21 older women composed the final sample and completed the second-day assessment.

This study consisted of two sessions of evaluation of the maximal isometric muscle strength of the trunk in asymptomatic elderly women, with a 48-hour interval between the test sessions.

Twenty-one physically inactive elderly women, who had not engaged in any activity or exercise program in the past three months, participated in the study. But they had already participated in a regular strength training protocol. The following inclusion criteria were adopted to select the participants: (a) age above 60 years, (b) no limiting back pain in the previous year, and (c) no medical or physiotherapeutic treatment for back pain in the previous year. We excluded from the present study the subjects who presented limitations for the tests and those who did not attend one of the evaluation sessions at the dates and times previously scheduled.

Prior to the evaluation sessions, the participants were instructed to avoid exercise during the previous 24 h. All subjects were informed about the study, and they provided their signed written informed consent in accordance with resolution 466/2012 of the National Commission of Ethics in Research of the National Health Council in agreement with the ethical principles expressed in the Declaration of Helsinki (1964, restated in 1975, 1983, 1989, 1996, 2000, 2008, and 2013) of the World Medical Association. This study was approved by the Committee of Ethics in Research with Human Beings of the Federal University of Sergipe (number: 060568/2017).

### 2.2. Protocol

The tests were carried out in the same place, administered in the same order, and supervised by the same researchers. Before the tests, the researchers adjusted the devices (according to the anthropometric characteristics of the participants) and instructed them about body positioning.

The body mass of the participants was measured in kilograms (kg) using a digital platform scale (Filizola 2002, São Paulo, SP, Brazil) calibrated from 0 to 150 kg and with a precision of 0.1 kg. The participants' height was measured with a stadiometer fixed to the wall (Sanny ES2040, São Bernardo do Campo, SP, Brazil), and the average of three measurements was recorded as the final result. Height measurements were recorded with a precision of 0.1 [14].

To evaluate the maximal isometric strength of the trunk muscles, a fixed seat platform with an adjustable support for hip and lower limbs was adjusted according to the height of each individual, in order to isolate the trunk muscles to perform the test. The subjects were placed sitting on the platform with an anterior pelvic tilt to avoid compensatory activation of the lower limbs. The legs were fastened to the seat platform by a Velcro strap [8, 15]. From this position, the muscle strength of trunk extensors and flexors was measured by a precalibrated digital loading cell (Kyoto, 333 A, Hown Dong, South Korea), connected to the MuscleLab software (Ergotest Innovation, Porsgrunn, Norway). A familiarization of the test was made for each condition (flexors/extensors). After the familiarization, three measures were performed with rest of 30 s between each one. If there was a discrepancy of more than 100 N in one of the three values, this measure was repeated. In addition, for statistical purposes, the average of the three measures was calculated. The values of the force in newton (N) and the rate of force development (RFD) in newton/second (N/s) were recorded.

**Figure 1:**
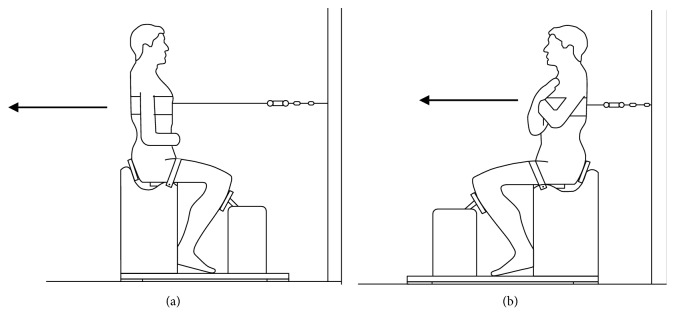
Side view during (a) extension and (b) flexion of the trunk.

To evaluate the extensors of the trunk, the participants were positioned with the trunk at 0° of flexion (Figure 1(a)). The load cell was attached to the wall by an adjustable tensioner and connected to the individual by a Velcro strap positioned at the level of the xiphoid process. From this positioning, a maximal isometric contraction was performed in the trunk extension. For the evaluation of the trunk flexors, the load cell was fixed to the wall behind the participant, with the strap attached to the trunk at the height of the scapula. From the initial position, the trunk flexion was performed to measure the maximum isometric force (Figure 1).

Participants performed a warmup that consisted of at least three submaximal slow dynamic motions throughout the range of trunk movement and performed 1 or 2 isometric contractions, according to the test protocol, at submaximal loads. Thereafter, volunteers generated their maximum isometric contraction by gradually increasing their torque moment up to their maximum within the first 2-3 s of each contraction. The entire test protocol was performed under the supervision of the previously trained examiner. The best value obtained out of 2 attempts was recorded. When a variation greater than 10% was observed between the two trials or when the peak force was reached after three seconds of maximal isometric action duration, retesting was performed until the test criteria were satisfied. The intervals between each trial were at least 15 s, and the flexor and extensor tests were separated by a resting period of 5 min. The instructions for conducting the test and the verbal stimuli of encouragement were standardized. The order of different muscle group tests was kept constant, with back extension tests first, followed by trunk flexion tests [10, 12].

### 2.3. Statistical Analysis

The normality of the data was assumed by the Shapiro–Wilk test. Descriptive analysis was performed with data presented as mean ± standard deviation and confidence interval (95%). Considered as of the parametric statistics, the comparison of the values for trunk muscle strength was performed by Student's *t*-test for dependent samples. The interclass correlation coefficient (ICC) was considered small (up to 0.25), low (0.26–0.49), moderate (0.50–0. 69), high (0.70–0.89), and very high (above 0.90), according to the description of the previous studies [16]; the coefficient of variation (CV) was considered optimal for values below 10% [9], and the graphical plots of Bland–Altman were used to verify the agreement between the measurements from the bias analysis and limits of agreement to 95% [17]. The standard error of measurement (SEM) was also calculated through the following equation: SEM = SD ∗ √(1 − ICC), where SD corresponds to the standard deviation. The minimum detectable difference (MDD) was calculated using the following equation: MDD = 1.96 × (SEM × √2).

For all analyses, the statistical significance considered was *p* < 0.05. Statistical procedures were performed using SPSS® software version 22.0.

## 3. Results

**Figure 2:**
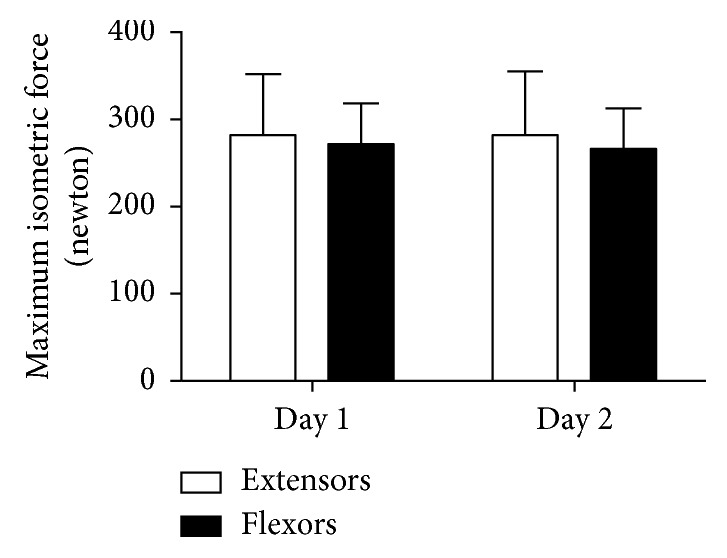
Values of the maximal isometric strength of the extensor and flexor muscles of the trunk obtained in the two days of evaluation (*n*=21). The data are expressed as mean ± standard deviation (SD).

The general characteristics of the 21 participants were 64 ± 4 years old, 70 ± 11 kg, and 154.0 ± 4.8 cm. No statistically significant differences (*p* > 0.05) were observed between test and retest in the maximal isometric force of the extensor (day 1 : 281.7 ± 69.7; day 2 : 281.8 ± 73.3) and flexor (day 1 : 271.1 ± 47.2; day 2 : 266.1 ± 46.6) muscles of the trunk (Figure 2).

**Figure 3:**
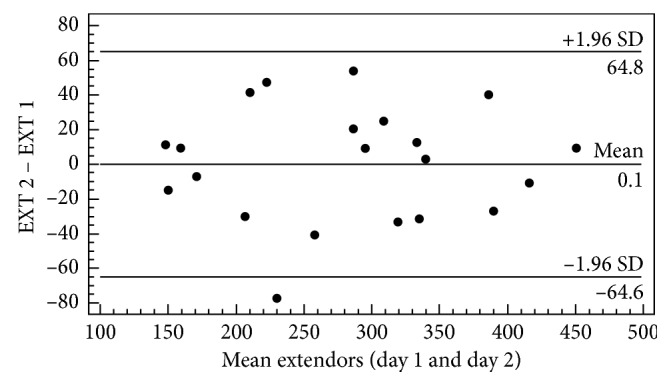
Graphical representation of Bland–Altman plot for visualization of the differences and averages between the first and second day of evaluation, obtained by the maximum strength (N) of the muscle trunk extensors (EXT) (*n*=21).

**Figure 4:**
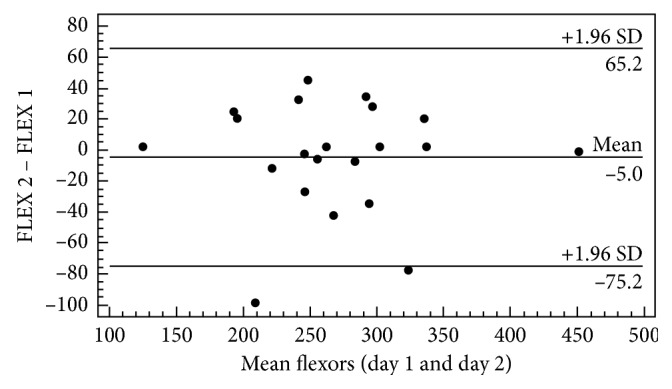
Graphical representation of Bland–Altman plot for visualization of differences and averages between the first and second day of evaluation, obtained by maximum strength trunk muscle flexor (FLEX) (*n*=21).

Thus, the calculations of the reliability indexes for the evaluated muscle assessments with ICC and CV were performed, followed by the calculation of the SEM and MDD for the values obtained between the first and second evaluation days (Table 1). The concordance analysis between the first and second testing days for the strength of the extensor and flexor trunk muscles is presented in Figures 3 and 4, respectively.

In Figure 3, when analyzing the extensor muscles, it was possible to observe a low dispersion of the points and that all the individuals are present within the acceptable limits of agreement, with the exception of only one participant.

Similarly, when analyzing the flexor muscles (Figure 4), it was possible to verify similar behavior where the points are close to the difference of the means and again all the individuals are within the limits of agreement, except for two of them.

## 4. Discussion

The reliability of the results of the muscular strength tests is crucial in order to accurately evaluate the performance [18]. The results of the present study indicated that the test protocol used to evaluate the maximal isometric strength of the trunk muscles in the elderly presents acceptable reliability considering the stabilization of the values measured in the test and retest.

The test was well tolerated by the study subjects, with no associated adverse events, which demonstrates that this evaluation protocol can be safely used for assessing trunk strength in the elderly. These results corroborate an investigation that verified the reliability of the isometric strength of the trunk in the elderly population [10].

The high reliability of the test, observed through high ICC and low CV and SEM, is presumably related to several factors, including the standardization of the instructions to the evaluated ones, the adoption of familiarization procedures, the adjustment of the fixed seat platform according to the size of the members of each individual, the fixed order of the tests, and the supervision of experienced evaluators. It should be noted that only two participants reported the sensation of muscle fatigue on the second day of evaluation, which did not affect their performance during the test.

For the ICC between the first and second day of evaluation in the trunk extensor (0.93) and flexor (0.86) muscles, we observed values classified as very high and high, respectively, according to the scale used by Jonson et al. [19]. In addition to the low coefficient of variation of trunk extensors and flexors between days (CV = 9%), the test used had good reliability for the tracking of measures for research [11]. To our knowledge, only two studies in the literature have investigated the reliability of the maximum isometric strength test in the elderly [1, 10]. However, measures of the absolute reliability of the evaluation instrument were not considered [1, 10]. In addition, most studies have investigated only the association between changes in trunk muscle strength and reduction in functional performance in the elderly, such as sit and stand tests, 6-minute walk, and Berg balance [2, 3, 20].

Thus, similar to the findings of this study, Roth et al. [18] compared the reliability of back muscle strength in isometric and isokinetic conditions and reported high reliability of both methods for trunk strength in youngsters. Despite investigating a homogeneous group of youngsters, the study demonstrates that isometric trunk strength is as reliable as an isokinetic condition. The study by Kienbacher et al. [10] who performed a test-retest for isometric strength of the trunk extensors and flexors in healthy individuals (>50 years old) observed ICC values ≥0.75 for both muscle groups. Thus, these measures had good reliability [10].

When analyzing the SEM of the instrument, low values ranged from 17.6 to 18.4% for the trunk extensors and flexors. A similar study, while examining the intraobserver reliability of the isometric trunk strength in subjects with chronic low back pain, indicated a high reliability of isometric dynamometry (0.93–0.97) and SEM that ranged from 26 to 51.7% for the strength of the back muscle flexors and extensors [5]. However, the population recruited in the aforementioned research cannot be compared to that in our study since the individuals with lower back pain have a distinct pattern of muscular recruitment, arising from the mechanism of the pain [21]. Alternatively, our findings are consistent when compared to the research by Kienbacher et al. [10] when analyzing the SEM for maximal isometric strength in women aged above 50 years, with values of 13.7 and 5.0% for the trunk extensors and flexors, respectively [10].

In addition to the reproducibility analysis, the MDD values were evaluated. According to Hopkins [11], the probability of finding the performance changes after an intervention depends on the absolute reliability; therefore, it becomes important to quantify the measurement error. Large variations of force in the repeated tests reduce the detection of changes over time. Thus, as many studies aim to detect the changes resulting from the interventions, it is important to adopt methods that allow the identification of minimal changes [11].

The MDD results of the trunk extensors (51.1 N) and flexors (48.9 N) of the test did not occur due to evaluation error. According to the data, a change value observed in a postintervention situation that is lower than the MDD is not distinguishable from the measurement error; it means that there was no change in the parameter evaluated. Similarly, if the value obtained is equal or above the values given in the table, this means that there was a true change in the maximum trunk strength assessed by the test. This study is the first one reporting absolute reliability statistics associated with maximal trunk strength tests in the older people; therefore, no comparison of these variables could be made. However, a similar study of maximal limb strength in older adults determined MDD for measurements of knee flexion and extension in individuals over 50 years old and observed an MDD between 46 and 79 N [22].

Considering the information obtained from the Bland–Altman plot, we observed uniform variability of mean performance for the two muscle groups tested, where the bias between the first and second day remained close to zero for a majority of the subjects. Therefore, the low dispersion observed results from the fact that all subjects presented values within the acceptable limits of agreement. Although a greater limit of concordance was observed for the measurement of the strength of the trunk flexor muscles, the outliers did not influence the homogeneous distribution of the point dispersion. Such a difference in distribution is common for measures of physical performance, which can be explained by both physiological and psychological phenomena [23].

Thus, it is important to emphasize that professionals need to be able to interpret the measurement changes of an evaluation instrument and consequently determine the effectiveness of different interventions. The test-retest studies provide information about relative reliability (ICC), that is, the degree to which the repeated measures reveal consistent classification of the individuals' scores within a group [24]. Absolute reliability measures (SEM and MDD) describe individual variability and measurement error. They are important in determining levels of clinically significant changes resulting from intervention processes [24]. Therefore, it is suggested that the fixed seating platform can be used to evaluate the isometric strength of the trunk muscles of the elderly, requiring only one evaluation session.

Our study has as a limitation regarding the extrapolation of data to force applied at other angles because it is an evaluation of isometric force. However, tests that evaluate different angulations, such as isokinetics, are usually costly. Thus, the main contribution of this study is to offer a simple and low-cost protocol that makes it possible to investigate a cause-and-effect relationship from different types of training, without the results being derived from a learning effect of the sample.

It is concluded that the test protocol for evaluation of the maximal isometric force of the trunk flexor and extensor muscles in elderly women presents high reliability. The reproducibility of the data in the test and retest, performed with an interval of 48 h between them, confirms the hypothesis of the authors regarding the consistency of the measuring instrument.

## Figures and Tables

**Table 1:** Values of the isometric dynamometry tests of back muscle extensors and flexors, between days 1 and 2 (*n*=21), followed by ICC, CV, SEM, and MDD values.

Variables	Day 1	Day 2	Days 1 and 2			
	Mean ± SD	Mean ± SD	ICC	CV	SEM	MDD
Extensors	281.7 ± 69.7 N (250.0–313.4)	281.8 ± 73.3 N (248.4–315.2)	0.93	8.9%	18.4	51.1
Flexors	271.0 ± 47.2 N (249.6–292.6)	266.0 ± 46.6 N (244.9–287.3)	0.86	8.9%	17.6	48.9

Data are expressed as mean ± standard deviation (SD). N: newton; ICC: interclass correlation coefficient; CV: coefficient of variation in %; SEM: standard error of measurement in newton; MDD: minimum detectable difference in newton. The values of 95% confidence interval (lower-upper) are shown in parentheses.

## Data Availability

The data used to support the findings of this study are available from the corresponding author upon request.
